# The Frequency of *DPYD* c.557A>G in the Dominican Population and Its Association with African Ancestry

**DOI:** 10.3390/pharmaceutics17010008

**Published:** 2024-12-24

**Authors:** Mariela Guevara, Carla González de la Cruz, Fernanda Rodrigues-Soares, Ernesto Rodríguez, Caíque Manóchio, Eva Peñas-Lledó, Pedro Dorado, Adrián LLerena

**Affiliations:** 1 Research and Development Department, Universidad Nacional Pedro Henríquez Ureña, Santo Domingo 10203, Dominican Republic; ernest2288@yahoo.es (M.G.); er18-2087@unphu.edu.do (E.R.); 2 Personalized Medicine and Mental Health Unit, University Institute for Bio-Sanitary Research of Extremadura, 06080 Badajoz, Spain; carla.gonzalezd@externos.salud-juntaex.es (C.G.d.l.C.); fernanda.soares@uftm.edu.br (F.R.-S.); elledo@unex.es (E.P.-L.); allerena@unex.es (A.L.); 3 Department of Pathology, Genetic and Evolution, Biological and Natural Sciences Institute, Universidade Federal do Triângulo Mineiro, Uberaba 38025-350, Brazil; caique.manochio@gmail.com; 4 Department of Genetics, Ecology and Evolution, Universidade Federal de Minas Gerais, Belo Horizonte 31270-901, Brazil

**Keywords:** fluoropyrimidines, *DPYD*, cancer, African ancestry, pharmacogenetics

## Abstract

**Background/Objectives:** Genetic polymorphism of the dihydropyrimidine dehydrogenase gene (*DPYD*) is responsible for the variability found in the metabolism of fluoropyrimidines such as 5-fluorouracil (5-FU), capecitabine, or tegafur. The *DPYD* genotype is linked to variability in enzyme activity, 5-FU elimination, and toxicity. Approximately 10–40% of patients treated with fluoropyrimidines develop severe toxicity. The interethnic variability of *DPYD* gene variants in Afro-Latin Americans is poorly studied, thereby establishing a barrier to the implementation of personalized medicine in these populations. Therefore, the present study aims to analyze the frequency of *DPYD* variants with clinical relevance in the Dominican population and their association with genomic ancestry components. **Methods:** For this study, 196 healthy volunteers from the Dominican Republic were genotyped for *DPYD* variants by qPCR, and individual genomic ancestry analysis was performed in 178 individuals using 90 informative ancestry markers. Data from the 1000 Genomes project were also retrieved for comparison and increased statistical power. **Results and Conclusions**: The c.557A>G variant (decreased dihydropyrimidine dehydrogenase function) presented a frequency of 2.6% in the Dominican population. Moreover, the frequency of this variant is positively associated with African ancestry (r^2^ = 0.67, *p* = 1 × 10^−7^), which implies that individuals with high levels of African ancestry are more likely to present this variant. HapB3 is completely absent in Dominican, Mexican, Peruvian, Bangladeshi, and all East Asian and African populations, which probably makes its analysis dispensable in these populations. The implementation of pharmacogenetics in oncology, specifically *DPYD*, in populations of Afro-Latin American ancestry should include c.557A>G, to be able to carry out the safe and effective treatment of patients treated with fluoropyrimidines.

## 1. Introduction

Since it was introduced in 1957, 5-fluorouracil (5-FU) and its oral prodrug, capecitabine, have been used in the treatment of different solid tumors, such as colorectal cancer, gastrointestinal, breast, etc. [1]. Of these, 10–30% of patients treated with standard doses of fluoropyrimidines have reported severe toxicity, causing deaths in 0.2–1% of cases [2,3,4]. The rate of fluoropyrimidine catabolism is the main determinant of side effects and is dictated by the enzyme dihydropyrimidine dehydrogenase (DPD), which is implicated in about 50% of cases of severe toxicity [5]. The enzymes of the uracil anabolic pathway convert 5-FU to its active metabolite, with the DPD enzyme (encoded by the *DPYD* gene) being the limiting enzyme of this pathway; as such, DPD deficiency is highly predictive of adverse toxicity [6]. Approximately 3–5% of the general population show reduced DPD activity, while 0.2% have complete DPD deficiency [7].

The use of pharmacogenetics for the prescription of fluoropyrimidines enables the identification of patients at risk of severe toxicity prior to treatment, allowing the initial dose of fluoropyrimidines to be adjusted. In particular, the *DPYD* risk variants (c.19051+1G>A, c.1679T>G, c.2846A>T and c.1236G>A, plus c.1129-5923C>G) are relevant for predicting the occurrence of this toxicity [8]. Most published pharmacogenetic studies on fluoropyrimidine toxicity and its relationship to the *DPYD* gene focus on a few variants [9], such as DPYD*2A and DPYD*13, which are associated with near-complete protein deficiency in homozygotes [10], and c.2846A>T (rs67376798) and HapB3 (c.1129-5923C>G + c.1236G>A), which are associated with a moderate loss of protein function [11]. Until now, the c.1129-5923C>G and c.1236G>A variants were considered to be in linkage disequilibrium in European populations [8]; however, recent studies show that this may not be true for all people and suggest not considering the linkage disequilibrium of these two variants in order not to erroneously predict decreased enzyme activity [12,13]. Although around 480 *DPYD* genetic variants have been identified, only 28 exert established effects on DPD activity [14,15]. The reporting of adverse effects associated with fluoropyrimidines has made possible further research on drug safety and the creation of clinical recommendations to prevent these adverse reactions in the European population [8,16]. However, the application of pharmacogenetics in other populations is still not standardized [17]. One potential source for this disparity in response may be the differences in 5-FU metabolism between African Americans and Europeans. A previous study demonstrated that DPD deficiency was three times more prevalent in African American patients than in White patients [18]. In addition, ancestry may also influence the presence of specific polymorphisms. A study revealed that six *DPYD* SNPs were only detected in the African American population (IVS2-69, c.525G>A, c.557A>G, IVS8-31, c.1358C>G, and c.1371C>T) [19]. Specifically, lower DPD enzyme activity has been observed in African Americans than in Europeans, due to a nonsynonymous variant in exon 6, c.557A>G (rs115232898), with a frequency of 3–5% [8]. This non-synonymous variant has only been found in the African American population and not in the European American group; 2–4% of African American individuals with reduced DPD activity presented this variant, suggesting that it may be a risk allele for 5-FU toxicity in individuals of African ancestry [1,5,19,20,21]. Based on these observations, the c.557A>G variant has been listed as one of the *DPYD* variants associated with an increased risk of 5-FU severe adverse events [22]. Moreover, a recent study of an Alabama cohort showed that of 38 individuals of African ancestry with an actionable *DPYD* genotype, 34 (89.5%) possessed the c.557A>G variant [23]. In this study, the population of the Dominican Republic is analyzed because no studies of these characteristics have previously been reported in the country and because it has a predominantly African and European molecular ancestry [24,25,26]. The *DPYD* genetic variants recommended in clinical guidelines and routinely analyzed in the European population are analyzed, as well as two *DPYD* genetic variants that decrease or inhibit DPD activity with a high frequency in African and Afro-descendant populations. The present study aims to study the frequency of *DPYD* variants with clinical relevance in the Dominican population.

## 2. Materials and Methods

### 2.1. Subjects

The study cohort included 196 healthy, unrelated Dominican students and staff, recruited from the “Universidad Nacional Pedro Henríquez Ureña” (UNPHU, https://unphu.edu.do, accessed on 1 December 2024) in Santo Domingo, Dominican Republic. None of the participants were immigrants, which applied to at least two of their previous generations; their ages ranged between 20 and 48 years old, and 64.2% were women. The study adhered to the principles outlined in the Declaration of Helsinki for human research and was approved by the *Consejo Nacional de Bioética en Salud* Ethical Committee (Cod. 018-2022). Written informed consent was obtained from all participants before sample collection.

### 2.2. Selection of Variants

Most of the clinically recommended *DPYD* variants are rare in the general population; however, due to the potent toxicity related to DPD null function, it is recommended to include in the analysis those *DPYD* variants with a frequency greater than 0.1% in the general population [12]. In this study, the variants c.19051+1G>A (rs3918290, also known as DPYD*2A), c.1679T>G (rs55886062, DPYD *13, p.I560S), c.2846A>T (rs67376798, p.D949V), and c.1236G>A plus c.1129-5923C>G (rs75017182, rs56038477, HapB3) were selected to be analyzed in the Dominican Republic because they are on the *DPYD* genotyping panel that is widely implemented in the clinic and exhibit high frequency in European populations. Moreover, the c.1024G>A (rs183385770) variant was analyzed for presenting a frequency of 0.3% in the African American population and its clinical relevance, associated with DPD deficiency [8]. Additionally, the c.557A>G (rs115232898) variant was included, which is exclusive to the African American population and causes reduced DPD activity function, increasing the risk of toxicity in 5-FU treatment in individuals of African descent, such as the Dominican population. This study follows the standard nomenclature of the Human Genome Variation Society [27].

### 2.3. Genotyping of DPYD

Saliva samples were collected on buccal swabs, and DNA was subsequently extracted with the QIAamp DNA blood kit (QIAGEN, Hilden, Germany) and phenol-chloroform-isoamyl alcohol. DNA quality and concentration were evaluated by spectrometry (NanoDropOne, Thermo-Fisher Scientific, Inc., Greenville, NC, USA).

Analysis of *DPYD* was performed using commercially available genomic DNA and Taqman^®^ assays (Table 1) (Applied Biosystems, Foster City, CA, USA). *DPYD* genotypes were assigned according to the presence of “key” SNPs associated with the alleles of interest. All assays were performed to include both negative (no DNA) and positive (heterozygous and/or homozygous) control samples. The positive controls were used from previous studies of our group. Plates were read with QuantStudio5 (Applied Biosystems). The following thermocycling conditions were applied: 10 min for initial denaturation at 95 °C, 40 denaturation cycles of 15 s at 92 °C, and annealing at 60 °C for 1 min. Allele discrimination was performed for 30 s at 60 °C.

The Clinical Pharmacogenetics Implementation Consortium (CPIC) classifies the phenotype predicted from the genotype into normal metabolizers (NMs) when an individual carries two normal-function alleles; intermediate metabolizers (IMs) when individuals carrying one normal-function allele plus one no-function allele or one decreased-function allele, or an individual carrying two decreased-function alleles; and poor metabolizers (PMs) when an individual exhibits two no-function alleles or one no-function allele plus one decreased-function allele [8].

### 2.4. Continental Ancestry Analysis

In this study, African, European, and Native American individual ancestry was estimated in 178 Dominican individuals by genotyping 90 ancestry informative markers (AIMs). The admixture values were inferred using the model-based method implemented in Admixture software (version 1.3.0) [28], assuming a tri-hybrid model (K = 3), as described previously [29].

Genotype data from the 90 AIMs were also downloaded from the 1000 Genomes project for all 26 populations, and the same tri-hybrid admixture analysis was performed for these individuals [30]. Then, genotypes of the seven *DPYD* SNPs from these individuals were also downloaded and inserted into the database to create a larger analysis dataset.

### 2.5. Data Analyses

The estimated sample size was calculated to be 105 individuals, which was sufficient to estimate, with a confidence level of 95% and a precision of ± 3 percentage units, a population percentage that is expected to be around 2.5% (according to previous studies, the variants to be analyzed vary from 0 to 5%) [8], performed by the GRANMO calculator Ver. 8.0 (https://www.datarus.eu/aplicaciones/granmo/, accessed on 1 September 2023). The allele and genotype frequencies of *DPYD* were calculated using the Adegenet package [31] and ancestry individual proportions were plotted with the *barplot* function in R Platform (www.r-project.org, accessed on 15 July 2024). To describe the dependence of *DPYD* allele frequencies on the three ancestry components, the linear coefficient beta, its significance (*p*-value), and the percentage of *DPYD* allele frequencies variance explained by each continental ancestry (r^2^) were estimated by a linear regression analysis using the *lm*() function in R platform (www.r-project.org, accessed on 15 July 2024). Linkage disequilibrium for HapB3 [c.1236G>A; c.1129-5923C>G] was calculated using Plink [32], with the —r2 flag.

## 3. Results

This is the first pharmacogenetic study of *DPYD* in the Dominican population, where the frequency of clinically relevant *DPYD* polymorphisms is analyzed. A total of 196 healthy individuals have been genotyped for seven *DPYD* variants (Table 1) that are relevant to clinical practice.

From 196 Dominican individuals genotyped for genomic ancestry, 12 were self-reported Whites (6.12%), 15 Afro-descendants (7.65%), and 169 Admixed (86.22%). The molecular ancestry analysis showed that this population is 23.8% European, 42.6% Native American, and 33.6% African. All *DPYD* variants with clinical recommendations implemented in the CPIC European gene panel were absent, except for c.2846A>T (0.3%). The c.1024G>A variant was present in one sample from the Dominican Republic (0.5%). Interestingly, the c.557A>G polymorphism, which reduces DPD activity and is not recommended by CPIC, presented a frequency of 2.6% in the Dominican population. Of the 10 individuals carrying c.557A>G (5.1% of the population studied), eight reported themselves as Admixed, two as White, and none as Afro-descendant.

According to the variants analyzed in this study, the frequency of IMs in the Dominican Republic is 5.6%. If the c.557A>G variant was not analyzed, as proposed by the CPIC panel, only 0.5% of the individuals would present an IM phenotype.

### 3.1. Association of DPYD Variants with Genomic Ancestry

The results of the linear regression analysis of *DPYD* variants’ frequencies and the three ancestry components of the 26 populations from the 1000 Genomes project and the Dominican Republic are shown in Table 2. HapB3 presented a positive association with European ancestry (r^2^ = 0.51; *p*-value < 0.0001), and, interestingly, c.557A>G was positively associated with African ancestry (r^2^ = 0.66; *p*-value < 0.0001).

**Figure 1:**
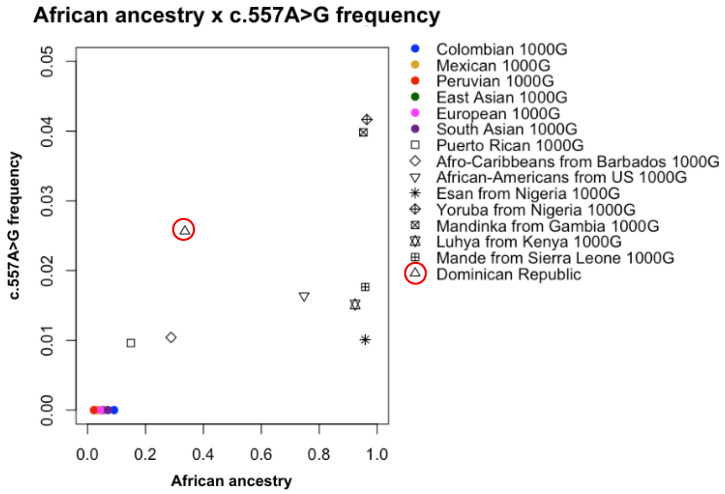
*DPYD* c.557A>G allele frequencies as a function of African ancestry proportions in the 1000 Genomes project populations, plus the Dominican Republic population. East Asian: CDX: Dai Chinese from Xishuangbanna, CHS: Han Chinese from the south, CHB: Han Chinese from Beijing, JPT: Japanese from Tokyo; European: TSI: Italians from Tuscany, IBS: Iberians from Spain, GBR: British from England and Scotland, CEU: European descendants from Utah, FIN: Finns from Finland; South Asian: PJL: Pakistanis, GIH: Gujarati Indians in Houston USA, ITU: Telugu Indians in the UK, STU: Sri Lankan Tamils in the UK, BEB: Bengalis from Bangladesh, and KHV: Kinh Vietnamese from Ho Chi Minh City.

In addition, Figure 1 shows the c.557A>G frequency as a function of African ancestry in the 1000 Genomes project populations and in the Dominican Republic. Our sample presents a less than 50% African ancestry, such as the other Caribbean populations of Puerto Ricans and Afro-Caribbeans from Barbados. However, its frequency of c.557A>G is higher than those of these neighbors and is also higher than that of some African populations, such as the Esan from Nigeria, Luhya from Kenya, and Mande from Sierra Leone.

### 3.2. HapB3 Linkage Disequilibrium

Previous studies have demonstrated that c.1236G>A and c.1129-5923C>G are in linkage disequilibrium (LD), and, for this reason, they were called Haplotype B3 (HapB3) [12,13]. However, the latest update of the CPIC Clinical Guideline (March 2024) recommends the analysis of both variants to determine the corresponding phenotype and to give a specific and safe recommendation, according to a recent study [13]. This issue is currently under discussion, so, for this reason, the LD r^2^ values are presented in this study for the Dominican Republic and all 1000 Genomes project populations separately (Supplementary Table S1). It was only possible to calculate the LD between c.1236G>A and c.1129-5923C>G for 13 of the 27 populations (all r^2^ = 1) because both variants are absent in the Dominican, Mexican, Peruvian, Bangladeshi, and all East Asian and African populations.

## 4. Discussion

*DPYD* substrate drugs are employed in the treatment of not only one of the most relevant diseases in general but also for low-income populations, such as the Afro-Latin American populations studied in this study and in a previous study in the Dominican Republic [29]. One of the major problems in global health is the availability of drugs for the treatment of these relevant diseases. This problem is compounded by the lack of information on the effect of drugs on indigenous and mestizo populations, especially in Latin America, particularly among Afro-Latin Americans. Implementing pharmacogenetics to determine variability can be very relevant for patients with cancer, but may also be relevant in other very important pathologies such as antidepressants, the risk of depression [33,34] or suicide [35], or treatment failure with antidepressants [36], antipsychotics [37,38], or antiepileptics [39].

Genetic variations in *DPYD* can affect the patient’s response to treatment with fluoropyrimidines during the treatment of various types of cancer and can lead to severe, even lethal, toxic reactions. Pharmacogenonics-based clinical guidelines (ex. CPIC) describe the dosage of recommended drug change for individuals with heterozygous genotypes of deleterious variants. The CPIC Clinical Guideline recommends reducing the initial dose of fluoropyrimidines by 25–50% and adjusting the dose, depending on the toxicity or therapeutic control of the drug in those patients who have decreased DPD activity (IM), while for those patients with a complete DPD deficiency (PM), and thus a risk of severe or even fatal toxicity, the guideline recommends avoiding the use of fluoropyrimidines [8]. Following this recommendation, in 5.1% of the Dominican population, the dose should be reduced by 25–50% to avoid adverse reactions due to the presence of c.557A>G, which is not currently included among the recommended variants [8].

One of the limitations of this study is the sample size (n = 196), although this is the first study to study *DPYD* variants in the Dominican population. Moreover, oncology patients are not analyzed, so clinical studies involving this population are needed.

The four variants with clinical relevance described in the CPIC guideline as causing decreased function and risk of toxicity are: c.1905+1G>A, c.1679T>G, c.2846A>T, and HapB3 (c.1236G>A; c.1129-5923C>G). Of these variants, c.19051+1G>A and c.1679T>G have the most deleterious impact on DPD activity, whereas c.2846A>T and c.1129–5923C>G result in moderately reduced DPD activity [8]. In this guideline, these variants were chosen due to their “population frequency and established impact on enzyme function and toxicity risk”, which is curious because HapB3 is completely absent in African and East Asian populations. However, in the European population, HapB3 is the variant most frequently associated with decreasing function (4.7%), followed by c.19051+1G>A (1.6%) and c.2846A>T (0.7%) [8]. In fact, this genetic panel of *DPYD* variants identifies less than 20% of patients as being at risk of severe treatment-related toxicity to fluoropyrimidines, if considering the whole world [10].

**Figure 2:**
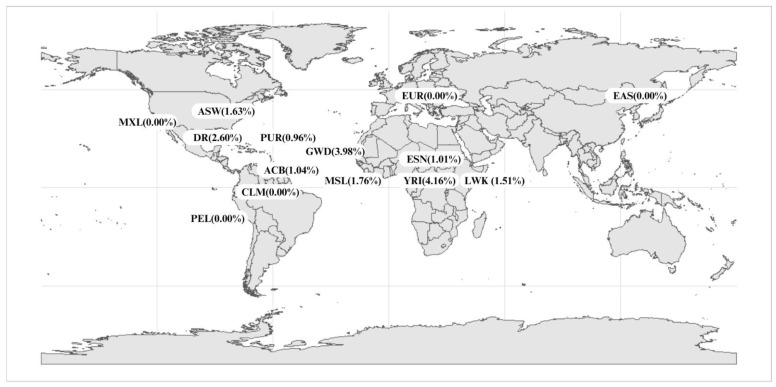
World map presenting the frequency of c.557A>G in 1000 Genomes project populations and in the Dominican Republic. EAS: Dai Chinese from Xishuangbanna, Han Chinese from the South, Han Chinese from Beijing, Japanese from Tokyo; EUR: Italians from Tuscany, Iberians from Spain, British from England and Scotland, European descendants from Utah, Finns from Finland; ESN: Esan from Nigeria, LWK: Luhya from Kenya, YRI: Yoruba from Nigeria, MSL: Mande from Sierra Leone, GWD: Mandinka from Gambia, ASW: African-Americans from the southwestern United States, MXL: Mexican descent from Los Angeles, ACB: Afro-Caribbeans from Barbados, CLM: Colombians from Medellín, PEL: Peruvians from Lima, DR: Dominican Republic (present study).

The c.557A>G polymorphism presents a frequency range of 1–4% in African populations ([29]; Supplementary Table S2; Figure 2), while it is practically absent in others. In the 1000 Genomes project populations, the c.557A>G variant frequency is 2.5% in African populations, reaching 4% among the Mandinka in the Gambia and the Yoruba in Ibadan, Nigeria, which share similar genetic traits and have their origins in the same geographic area of West Africa (Figure 2) [30]. More specifically, the frequency of this variant (Table 3) is higher in Sub-Saharan Africans (2.6%) than in African Americans/Afro-Caribbeans (average 1.3%); the low frequency in Somalis likely reflects their distinct ancestry from West Africans [40,41]. Compared to the population of the Dominican Republic, the Puerto Rican population presents less African ancestry (20%) due to its history. These individuals exhibit more Native American and European components but, even so, the c.557A>G variant is present in this population, which is different from other Latino populations living in the continent, such as Mexicans, Peruvians, and Colombians, in whom the variant is absent (Figure 1 and Figure 2). Afro-Caribbeans from Barbados have 28.8% of African ancestry, which is less than Afro-Americans from the USA (75%). Even so, both populations present similar frequencies of the c.557A>G variant. This may be due to an association among chromosome African ancestry tracts in this region.

Ancestry genomic mapping studies suggest that Kenyan individuals have a West African ancestral component, whereas individuals from Somalia or Ethiopia present a Middle Eastern and European ancestral component [42]. This distinction among the ancestral components could explain the high frequency of c.557A>G in Kenya compared to the Somali population [1]. African Americans are estimated to have an average West African ancestry of 50–77% [43], conferring a high probability of finding c.557A>G in those populations. These findings could be compared with the results of our study, suggesting that our population may have a West African ancestral component due to the high frequency (2.6%) of the c.557A>G variant.

This variant has also been observed in the Puerto Rican population (<1%) from the 1000 Genomes project, which presents 15% of African ancestry (Figure 1). Despite the low percentage of African ancestry in this population, their Dominican Republic neighbor may also need the c.557A>G analysis in *DPYD* pharmacogenomic implementation.

This variant reduces the enzymatic function of DPD, leading to potentially severe and even lethal adverse reactions that are associated with fluoropyrimidine treatment for many types of cancer. However, there are still very few published clinical studies considering this variant [21].

According to CPIC, the c.1024G>A variant has a frequency of 0.3% in African American/Afro-Caribbean populations and 0% in Sub-Saharan African populations [8]. In all 1000 Genomes project populations, except for Afro-Americans, this variant is absent (Supplementary Table S2). These frequencies support our results, wherein the frequency found in the Dominican population is 0%. This is another indication of the possible West African ancestral component of our population.

Besides these findings, our regression analysis shows a positive association between African ancestry and the frequency of c.557A>G. In other words, the more African ancestry an individual possesses, the higher the probability of exhibiting the c.557A>G variant. Additionally, Dominicans present a higher frequency of this variant than other Latin, Caribbean, and African populations (Figure 2), showing that the analysis of this variant in this population is crucial for *DPYD* pharmacogenetics implementation. The IMs in the Dominican Republic (5.1%) were identified only because of the genotyping of the c.557A>G variant, which is not included in the CPIC panel. This means that pharmacogenetic implementation would fail in 549,473 people (5.1%) of the Dominican population) if the CPIC panel was chosen [44].

Furthermore, the LD analysis of both HapB3 variants was not possible to calculate for 14 of the 27 populations, due to the absence of both variants in the Dominican, Mexican, Peruvian, Bangladeshi, and all East Asian and African populations. Therefore, testing the presence of the c.557A>G variant is much more important in African and Afro-descendant populations than HapB3.

The implementation of a genetic panel of *DPYD* variants with high frequency and clinical relevance in African American populations is essential to provide the efficient and safe treatment of fluoropyrimidines in different types of cancer. Many clinical cases show that the genotyping of toxicity-related variants leads to a change in the occurrence of adverse reactions [45].

International regulatory agencies establish many levels of recommendation for *DPYD* analysis. The Swissmedic agency requires that *DPYD* analysis be conducted before fluoropyrimidine treatment, but does not specify which variants should be analyzed [46]. The Health Canada (Santé Canada) group recommends *DPYD* analysis without specifying any variant [47]. The European Medicines Agency has recommended the genetic test of *DPYD* since June 2020 to analyze those variants with high frequency in the European population (c.1905+1G>A, c.1679T>G, c.2846A>T and c.1236G>A; c.1129-5923C>G), commenting that these variants are absent in African, Afro-American and Asian populations [48]. The Food and Drugs Administration also recommends the analysis of these four *DPYD* variants before fluoropyrimidine treatment. However, this agency mentions that c.557A>G is found in individuals of African ancestry, even though its analysis is not recommended [49]. The AEMPS (Spanish Agency of Medicines and Health Products) has recently published its database of pharmacogenomic biomarkers, recommending the analysis of *DPYD* for correct prescription in treatments with fluoropyrimidines; however, like the rest of the regulatory agencies, only the analysis of the four European variants is recommended [50,51].

Despite the importance of understanding inter-ethnic and inter-individual variability in drug response, there are few studies on the genetic polymorphisms of drug metabolization and transporter and receptor enzymes in the various ethnic groups in Latin America, and the frequency of the different phenotypes is unknown. An additional problem is the lack of ancestry markers for the assessment of different degrees of interbreeding. Therefore, in a previous study, we analyzed the frequency of the genetic polymorphisms of drug-metabolizing enzymes in the Afro-Latin American population of the Dominican Republic [29] and in Nicaragua [52], Ecuador [53], and Mexico [54]. Additionally, the existence of ‘phenocopies’ [55] due to the presence of other drugs and/or products of cultural habits should be taken into consideration [56]. Considering ethnicity and interactions with products of traditional medicine as a determinant of variability has been one of the principles of the Merida T’Ho Declaration of the RIBEF, in collaboration with CIOMS [57].

## 5. Conclusions

The *DPYD* pharmacogenetic test for non-European populations, especially Africans and Afro-descendants, must include the c.557A>G variant, as it reduces DPD activity and occurs frequently in these populations. Moreover, HapB3 is less important in these populations, as it is absent in Africa and in most of the Afro-descendant populations.

## Figures and Tables

**Table 1:** Description of the *DPYD* variants identified by RT-PCR.

*DPYD* Allele	rs ID	Nucleotide Change *	DPD Activity **	AA Change	Taqman Assay ID
*2A	rs3918290	c.1905+1G>A	None	NA	C__30633851_20
*13	rs55886062	c.1679T>G	None	p.I560S	C__11985548_10
HapB3	rs75017182	c.1129-5923C>G	Decreased	NA	C_104846637_10
	rs56038477	c.1236G>A		NA	C__25596099_30
NA	rs67376798	c.2846A>T	Decreased	p.D949V	C__27530948_10
NA	rs115232898	c.557 A>G	Decreased	p.Y186C	C_165900856_10
NA	rs183385770	c.1024 G>A	None	p.D342N	C_178512476_10

* According to the NCBI Reference Sequence NM_000110.4; ** According to PharmVar Consortium (https://www.pharmvar.org/gene/DPYD, accessed on 1 December 2024). Abbreviations: RT-PCR: real-time polymerase chain reaction; NA: not applicable; HapB3: haplotype-B3.

**Table 2:** Linear regression results considering the 26 populations from the 1000 Genomes project plus a Dominican Republic population (n = 178), tri-hybrid ancestry, and the frequency of *DPYD* variants.

Ancestry	Values	c.2846A>T	c.1905+1G>A	c.1679T>G	c.1236G>A; c.1129-5923C>G	c.1024G>A	c.557A>G
NAT	Beta	−0.003848	−0.002388	−0.0003407	−0.012862	−0.0007768	−0.016093
	*p*	0.1764	NS	NS	0.1069	NS	0.02461
	Adjusted r^2^	0.03475	0.00626		0.06467		0.1537
EUR	Beta	0.006351	0.03689	0.000762	**0.02892**	−0.000627	−0.017402
	*p*	0.01789	0.1293	0.1909	**1.62 × 10^−5^**	NS	0.01193
	Adjusted r^2^	0.1726		0.03012	**0.5125**		0.1964
AFR	Beta	−0.00224	−0.0033	−0.0004	−0.0139	0.001151	**0.0275329**
	*p*	NS	0.2392	NS	0.04852	0.1834	**1.32 × 10^−7^**
	Adjusted r^2^		0.01716		0.1127	0.03245	**0.6653**

NAT: Native American ancestry; EUR: European ancestry, AFR: African ancestry; NS: not significant.

**Table 3:** Allele frequency of *DPYD* variants in the Dominican Republic population (n =196) and other populations, as described in the *DPYD* CPIC guideline [8].

Frequencies of *DPYD* Variants in Biographical Groups							
*DPYD* Variant	Dominican Republic	Latino	African American/Afro-Caribbean	Sub-Saharan African	Central/South Asian	East Asian	European
c.557A>G	0.026	0.0012	0.0123	0.0259	0	0	0
c.1024G>A	0	0	0.0031	0	0	0	0
c.1236G>A; c.1129-5923C>G	0	0.0059	0.0031	0	0.0196	0	0.0237
c.1679T>G	0	0	0	0	0	0	0.0005
c.1905+1G>A	0	0.0007	0.0031	0	0.0054	0	0.0079
c.2846A>T	0.003	0.002	0.0031	0	0.0006	0	0.0037

## Data Availability

The raw data supporting the conclusions of this article will be made available by the authors on request.

## Supplementary Materials

The following supporting information can be downloaded at: https://www.mdpi.com/article/10.3390/pharmaceutics17010008/s1, Table S1: Linkage disequilibrium results for c.1129-5923C>G and c.1236G>A in 1000 Genomes project populations; Table S2: Allele frequencies of DPYD variants in 1000 Genomes project populations.
